# Production of virus-like particles with AsCas12a nuclease and CMV-driven crRNA for mammalian genome editing

**DOI:** 10.3389/fgeed.2026.1735339

**Published:** 2026-06-29

**Authors:** Natalia A. Kruglova, Sofiia E. Borovikova, Mikhail V. Shepelev

**Affiliations:** Center for Genome Research, Institute of Gene Biology Russian Academy of Sciences, Moscow, Russia

**Keywords:** AsCas12a, CRISPR/Cas delivery, genome editing, ribonucleoprotein complexes, virus-like particles

## Abstract

CRISPR/Cas genome editing tools represent a promising technology for biomedicine with significant therapeutic potential for numerous human diseases. However, efficient delivery of these tools into primary cells, particularly in the form of ribonucleoprotein (RNP) complexes, remains a critical bottleneck that limits clinical translation. Virus-like particles (VLPs) derived from human immunodeficiency virus type 1 (HIV-1) or murine leukemia virus (MLV) have emerged as promising delivery vehicles for RNP complexes, yet their activity is limited by suboptimal nuclease and guide RNA packaging. Previously, we generated NanoMEDIC VLPs incorporating the AsCas12a nuclease with CMV-driven crRNA, which demonstrated substantially enhanced editing efficiency over SpCas9-VLPs with U6-driven gRNA. Here, we describe a detailed protocol for a small-scale production of AsCas12a-VLPs using three distinct transfection methods [cationic lipids, polyethyleneimine (PEI), and calcium-phosphate] and a large-scale production of VLPs using calcium-phosphate transfection. We show that both production scales yield comparable nuclease loading into VLPs and similar editing efficiencies, reaching up to 60% of *CXCR4* knockout in Jurkat T cells.

## 1 Introduction

The clinical application of CRISPR/Cas genome editing in biomedicine faces a critical bottleneck: inefficient delivery of editing components to target cells (Wang and Doudna, 2023). Among various delivery strategies under investigation (Glass et al., 2018), virus-like particles (VLPs) derived from human immunodeficiency virus type 1 (HIV-1) or murine leukemia virus (MLV) have emerged as particularly promising vehicles for delivering genome editors (Choi et al., 2016; Lyu et al., 2019; Mangeot et al., 2019). These VLPs self-assemble from viral structural proteins and encapsulate CRISPR/Cas gene editing tools as ribonucleoprotein (RNP) complexes. VLPs offer several key advantages: they efficiently transduce diverse cell types, lack viral genomes, thereby minimizing integration risks, and enable transient nuclease expression that reduces off-target editing (Kim et al., 2014; Banskota et al., 2022). Additionally, VLPs can be directed to specific cell populations through pseudotyping with alternative envelope glycoproteins, enabling targeted RNP delivery (Mangeot et al., 2019; Gutierrez-Guerrero et al., 2021).

Multiple VLP systems employing distinct RNP packaging mechanisms have been engineered based on HIV-1 or MLV (Mazurov et al., 2023). Although direct comparisons between VLP types remain limited, evidence from independent studies suggests that the most effective designs incorporate RNPs through fusion of Cas9 to the Gag polyprotein, and guide RNA is co-packaged through its intrinsic interaction with the nuclease (Hamilton et al., 2021; Banskota et al., 2022). An alternative approach implemented in NanoMEDIC VLPs relies on chemically induced dimerization between FRB-tagged Cas9 and FKBP12-tagged Gag in the presence of AP21967 (Gee et al., 2020).

Recent years have witnessed continued innovation in VLP design, yielding increasingly sophisticated delivery platforms (An et al., 2024; Hamilton et al., 2024; Gutierrez-Guerrero et al., 2025; Halegua et al., 2025). However, most of the original research articles lack detailed technical information on VLP production. So far, only three step-by-step protocols have been published (Watanabe et al., 2023; Lu et al., 2025; Yoon et al., 2026). Watanabe et al. (2023) provided the workflow for the production of NanoMEDIC SpCas9-VLPs (Gee et al., 2020) that included both standard laboratory-scale production and large-scale manufacturing using flow electroporation technology. The second protocol describes the production of eVLPs containing base and prime editors that have been recently developed (Banskota et al., 2022; An et al., 2024) and briefly outlines methods for eVLP characterization (Lu et al., 2025). The most recent protocol depicts eVLP production specifically for editing mouse zygotes and embryos (Yoon et al., 2026), which was initially reported by the same group for mouse model generation (Jeong et al., 2025). Across the studies, VLP production protocols vary considerably in transfection methods, harvesting timepoints, concentration and purification procedures, transduction protocols, and other critical parameters.

**FIGURE 1:**
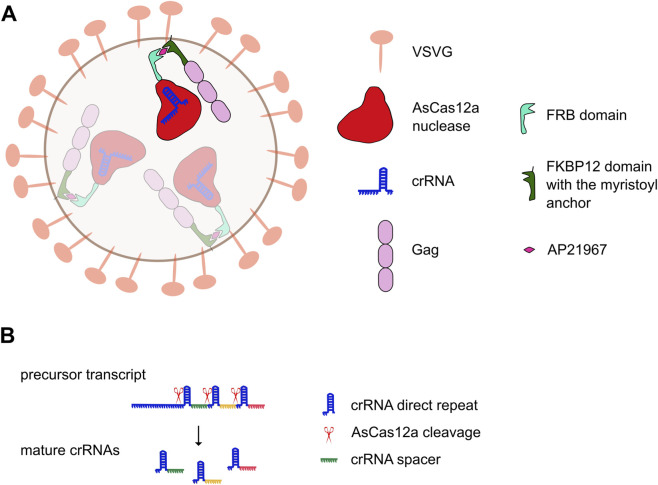
AsCas12a VLPs. **(A)** The packaging mechanism of NanoMEDIC VLPs. FRB-AsCas12a is recruited into VLPs via the interaction with FKBP12-Gag in the presence of a dimerizer molecule AP21967. **(B)** AsCas12a nuclease can process the pre-crRNA transcript. crRNAs are expressed as part of the Pol II (CMV)-promoter transcript individually or as an array with spacers flanked by direct repeats. AsCas12a cleaves the precursor transcript at direct repeat sequences and produces mature crRNAs (Campa et al., 2019). A transcript with up to six identical spacers was successfully used in our study (Borovikova et al., 2024). In this study, we used a plasmid coding for six identical spacers against the *CXCR4* gene.

Previously, we developed NanoMEDIC VLPs incorporating the AsCas12a nuclease with CMV promoter-driven crRNA. These VLPs demonstrated superior editing efficiency compared to SpCas9-VLPs and AsCas12a-VLPs produced with U6-driven crRNA (Figure 1A) (Borovikova et al., 2024). (Throughout this protocol, AsCas12a guide RNA is referred to as crRNA, SpCas9 guide RNA is referred to as gRNA, while ‘guide RNA’ is used when both nucleases are applicable.) We selected AsCas12a as a promising SpCas9 alternative due to its distinct Protospacer Adjacent Motif (PAM) requirements and cutting profile and, crucially, its intrinsic RNase activity that enables processing of pre-crRNA transcripts into mature crRNAs (Figure 1B) (Zhong et al., 2017; Safari et al., 2019). This self-processing capability allowed us to express crRNA from a Pol II (CMV) promoter, directing crRNA transcripts into the cytoplasm where VLPs assemble—a strategy that markedly enhanced VLP editing efficiency. This straightforward mechanism cannot be directly applied to SpCas9, which lacks intrinsic RNase activity.

**FIGURE 2:**
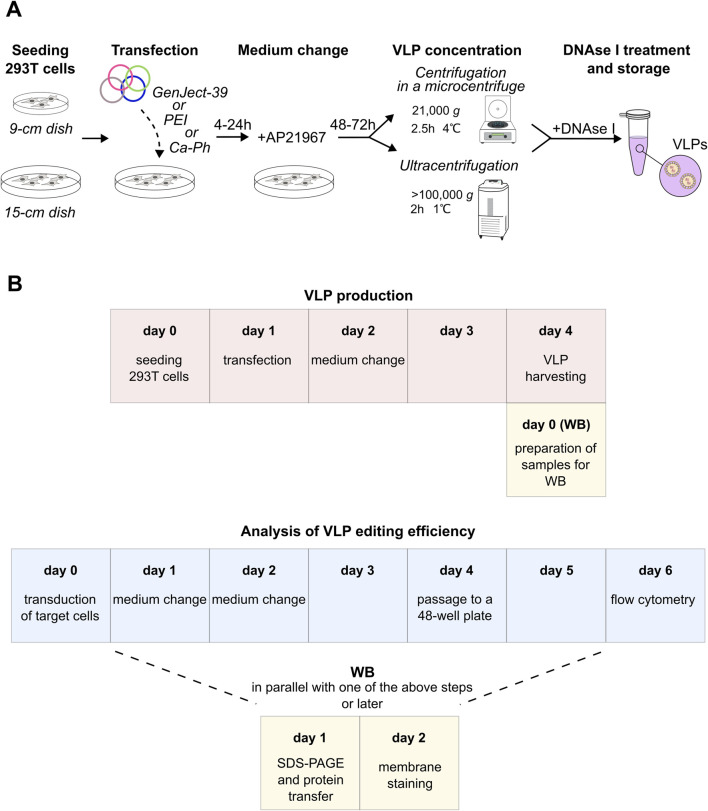
The scheme of VLP production **(A,B)** its timeline with VLP analysis. Images from Bioicons: “centrifuge” licensed under CC BY 3.0, “centrifuge-big” licensed under CC BY 4.0 and “microtube-closed-translucent” licensed under CC BY 3.0.

Here, we present a detailed step-by-step protocol for producing AsCas12a-VLPs with CMV-driven crRNA and their application for *CXCR4* knockout in Jurkat T cells (Figures 2A,B). The protocol encompasses VLP production using three distinct transfection methods and two approaches for concentrating VLPs, as well as production at two different scales. Example experiments include Western blot (WB) analysis to quantify AsCas12a content in VLP preparations and evaluation of VLP editing activity using flow cytometry analysis of the *CXCR4* gene knockout in transduced target cells. From initial seeding of producer cells, the complete protocol can be executed in approximately 12–19 days (Figure 2B). Although these VLPs do not contain a reporter transgene, their transduction efficiency can be monitored via the fluorescent protein mClover, which is passively incorporated into particles. By replacing plasmids, this protocol can be adapted to other VLP packaging systems and VLPs with alternative nucleases including SpCas9-VLPs, although different strategies for guide RNA expression would be required for SpCas9.

## 2 Materials and equipment

### 2.1 Materials

#### 2.1.1 Cell culture

- VLP producer cells: human embryonic kidney 293T cells (Cellosaurus CVCL_0063)
- Target cells: Jurkat T cells (Cellosaurus CVCL_0065)
- Full culture medium: DMEM/F12 (PanEco, Russia) supplemented with 10% fetal bovine serum (#SV30160.03, HyClone, USA), 4 mM L-glutamine (PanEco, Russia), and 10 μg/mL gentamycin (PanEco, Russia)
- Phosphate-buffered saline (PBS): 0.137 M NaCl, 2.7 mM KCl, 10 mM Na_2_HPO_4_, and 1.8 mM KH_2_PO_4_; pH 7.4.
- Trypsin–EDTA 0.05% solution (PanEco, Russia)

#### 2.1.2 Plasmids

- pCMV-FRB-AsCas12a (Borovikova et al., 2024)
- pHLS-FKBP12-Gag [#138476, Addgene (Gee et al., 2020)]
- pCMV-VSVG [#8454, Addgene (Stewart et al., 2003)]
- pCMV-mClover-6X4 [(Borovikova et al., 2024), the name pCMV-mClover-trpl-DR-cr6X4-DR was used in that study]

#### 2.1.3 Transfection

- Opti-MEM medium (#31985070, Thermo Fisher Scientific, USA)
- GenJect-39 (Molecta, Russia)
- Polyethyleneimine (PEI) (#408727, Sigma, USA)—1 mg/ml solution in deionized water, pH 7.0

Sterilize by filtration through a 0.2 µm PES filter, prepare 1 mL aliquots in 1.5 mL tubes, and store at −20 °C.

- 2× HBS: 50 mM HEPES (pH 7.1), 280 mM NaCl, and 1.5 mM Na_2_HPO_4_

After dissolution, adjust the pH with HCl. Prepare 6 aliquots of the buffer with pH values ranging from 6.95 to 7.20 at 0.05 intervals. Sterilize all solutions by filtration through a 0.2 µm PES filter and test them in transfection. Choose the best solution (pH 7.05 in our study), prepare 1 mL aliquots in 1.5 mL tubes, and store at −20 °C.

- 2M CaCl_2_ prepared in deionized water

Sterilize by filtration through a 0.2 µm PES filter, prepare 1 mL aliquots in 1.5 mL tubes, and store at −20 °C.

#### 2.1.4 Chemicals

- AP21967 (#635055, Takara Bio Inc., Japan) (1000× solution)
  Prepare 1000× stock solution of AP21967. Dissolve AP21967 in ethanol [used in this study as suggested by Watanabe et al. (2023)] or in DMSO to a final concentration of 300 μM. Aliquot and store at −70 °C.

#### 2.1.5 VLP concentration

- 20% sucrose (#H-1807–0.5, Panreac, Spain)/PBS solution
  Sterilize by filtration through a 0.2 µm PES filter and store at 4 °C.
- DNAse I (#M0303S, New England Biolabs, USA)
- 10× DNAse I buffer (#B0303S, New England Biolabs, USA)

#### 2.1.6 Flow cytometry

- PBS
- Labeled anti-CXCR4 antibody (#E-AB-F1157D, Elabscience, China)

Alternatively, use a primary mouse monoclonal antibody against CXCR4 (#sc-12764, clone 12G5, Santa Cruz Biotech-nology, USA) with secondary goat anti-mouse IgG antibodies conjugated with Alexa488 or Alexa546 (#A11001 and #A11003, respectively, Thermo Fisher Scientific, USA).

#### 2.1.7 Cell lysis

- Lysis buffer: 50 mM Tris-HCl, pH 8.0, 150 mM NaCl, 5 mM EDTA, 1% (w/v) Triton X-100, and 0.02% sodium azide
- 100 mM phenylmethylsulfonyl fluoride (PMSF, #3406.0005, Dia-M, Russia) stock solution in isopropanol.

#### 2.1.8 SDS-PAGE

- 4× SDS-PAGE sample buffer: 250 mM Tris-HCl, pH 6.8, 40% glycerol, 8% SDS, 4% 2-mercaptoethanol, and 0.2% bromphenol blue
- Resolving gel for Laemmli SDS-PAGE: 0.375 M Tris-HCl, pH 8.8, 7.5% acrylamide/bis-acrylamide solution (Т = 30%, С = 2.75%), 0.1% SDS, 0.05% ammonium persulfate, and 0.08% (v/v) TEMED
- Stacking gel for Laemmli SDS-PAGE: 0.125 M Tris-HCl, pH 6.8, 5% acrylamide/bis-acrylamide solution (Т = 30%, С = 2.75%), 0.1% SDS, 0.05% ammonium persulfate, and 0.04% (v/v) TEMED
- Laemmli electrophoresis running buffer: 0.025 M Tris-HCl, 0.192 M glycine, and 0.0035 M SDS
- Pre-stained protein markers (8–200 kDa, #G2083-250 μL and 55–320 kDa, G2085-250 µL Servicebio, China)

#### 2.1.9 Western blotting

- PVDF membrane with 0.45 µm pore size (#IPVH00010 Immobilon P, Merck Millipore, USA)
- Blotting filter paper (#G6001-200, Servicebio, China)
- Blocking buffer: 5% dry skimmed milk (#3905.0500, GOST 33629–2015, Russia)/0.1% Tween-20/PBS
- anti-HA rabbit monoclonal antibody (clone C29F4, #3724, Cell Signaling, USA)
- anti-α-tubulin mouse monoclonal antibody (clone 12G10, Sorbent, Russia)
- anti-p17 (Gag) mouse monoclonal antibody (p17 HIV Type 1, clone 32/5.8.42, #0801005, Zeptometrix, USA)
- Horseradish peroxidase-conjugated goat polyclonal antibodies against rabbit IgG (#7074, Cell Signaling, USA) or mouse IgG (#7076, Cell Signaling, USA)
- Chemiluminescent HRP substrate (#WBKLS0500 Immobilon, Merck Millipore, USA)

### 2.2 Accessories

All plastic used for VLP production should be sterile. Sterile plastic is not required for flow cytometry analysis (round-bottom 96-well plates) and Western blotting (tubes for samples and antibody dilution).

- 1.5 mL microcentrifuge tubes (#Ac-ACT-017-B, Accumax, India)
- 15 mL tubes (#EP-1500-J, Servicebio, China)
- 50 mL tubes (#EP-5000-J, Servicebio, China)
- 6-cm Petri dish (#20060, SPL, South Korea)
- 9-cm Petri dish (#20100, SPL, South Korea)
- 15-cm Petri dish (#20150, SPL, South Korea)
- Flat-bottom 96-well plate (#30096, SPL, South Korea)
- 48-well plate (#30048, SPL, South Korea)
- Round-bottom 96-well plate (#34096, SPL, South Korea)
- 10 mL syringe (#Rm073-3/534 203, Medpolymer, Russia)
- 0.45 μm PVDF syringe filter (#FPV403025, Jet Bio-Filtration, China, or equivalent, CA or PES syringe filters can be used as well)
- Pipette tips (10, 200, and 1,000 μL—#RS10-96, #RS200-96, and #RS1000-96, Genfollower, China)
- Pipettes (10, 50, 200, and 1,000 µL—Sartorius Biohit, Finland)
- Serological pipettes (5 and 10 mL—#SP-5-SF and #SP-10-SF, Servicebio, China)
- 8-channel pipette (30–300 μL, Sartorius Biohit, Finland)
- Serological pipette controller (Sartorius Biohit, Finland)
- Open-top tubes for ultracentrifuge (#355631, Beckman Coulter, USA)
- Small plastic containers

### 2.3 Equipment

- Centrifuge for 1.5 mL tubes (#5424, Eppendorf, Germany)
- Centrifuge for 15 and 50 mL tubes (Z32 6K, Hermle, Germany)
- Centrifuge for 96-well plates (KH20R-II, Kaida, China)
- Spectrophotometer Nano-500 (#AS-11060–00, Allsheng)
- Ultracentrifuge (Optima XPN-100, Beckman Coulter, USA) with the SW 32Ti rotor
- CO_2_ incubator (HERAcell VIOS 160i, Thermo Scientific, USA)
- Laminar flow hood (BMB-II- « Laminar-С»-1,5 NEOTERIC, Lamsystems, Russia)
- Tissue culture microscope (Micromed, Russia)
- Epifluorescent microscope (Nikon Eclipse TE2000-S, Japan)
- Cell counter with slides (C100-SE, RWD, China) or Goryaev chamber
- Heating block (#TDB-120, Biosan, Latvia)
- Equipment for protein electrophoresis (Mini-PROTEAN Tetra Cell, Bio-Rad, USA)
- Shaker (#Bio RS-24, Biosan, Latvia)
- Trans-Blot Turbo system (Bio-Rad, USA)
- ChemiDoc MP imaging system (Bio-Rad, USA)
- Aspirator (#FTA-1, Biosan, Latvia)
- Flow cytometer Cytoflex S with violet, blue, yellow, and red lasers and CytExpert 2.0 software (Beckman-Coulter, USA)

## 3 Methods

*Caution*: Although VLPs do not contain viral genetic material and cannot replicate, they can infect human cells causing editing of target genes and thus should be handled with necessary precautions.

- All work with VLPs is performed following BSL-2 practices in a class 2 type 2A biological safety cabinet while wearing personal protective equipment, including a laboratory coat and gloves.
- All equipment and liquids containing VLPs are disinfected with chlorine or ethanol before discarding.
- For VLP concentration, syringes are used without needles.
- For WB, VLPs are mixed with sample buffer, heated, and can then be handled at the bench.

### 3.1 Protocol outline

#### 3.1.1 VLP production mechanism

**FIGURE 3:**
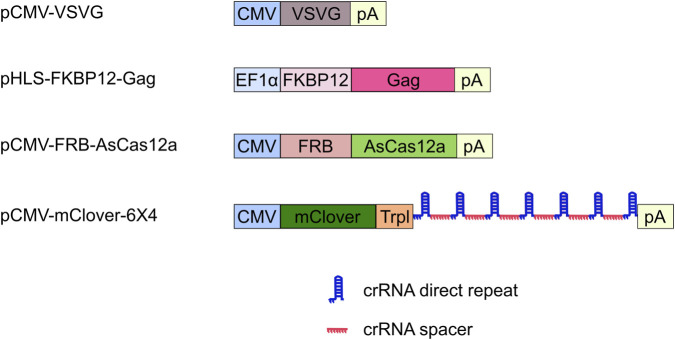
Plasmid constructs used for VLP production.

AsCas12a-VLPs are produced by transfecting 293T cells with four plasmids (Figure 3).

1. The envelope plasmid encodes the VLP surface protein that mediates receptor binding and membrane fusion (in some cases, these functions are performed using two separate proteins). VSVG is used in most cases.
2. The packaging plasmid encodes viral structural proteins needed for VLP assembly.
3. The plasmid that codes for a genome editor (AsCas12a) with a packaging domain (FRB in the NanoMEDIC system).
4. The plasmid that encodes crRNA. For better flexibility, we use two separate plasmids encoding AsCas12a and crRNA.

VLP assembly takes place in the cell cytoplasm under the plasma membrane, where the viral Gag polyprotein self-assembles and recruits the nuclease through a packaging domain (Mazurov et al., 2023). In the case of NanoMEDIC VLPs, FKBP12-Gag self-assembles while anchored to the plasma membrane via the myristoyl moiety (Gee et al., 2020). The FRB-fused AsCas12a is recruited into the forming particles in the presence of a dimerizing molecule AP21967 that is added to the medium after transfection. crRNA is packaged in a complex with FRB-AsCas12a. The envelope protein VSVG present on the plasma membrane covers the VLP surface. Thus, relying on viral mechanisms, VLPs assemble and then bud from the plasma membrane. The cell culture supernatant is then collected, and VLPs are concentrated. The resultant preparation is used to transduce target cells. VSVG binds a low-density lipoprotein receptor (LDLR) and initiates fusion after the endosomal uptake of VLPs. The viral core is released into the cytoplasm. FKBP12-Gag is bound to the membrane; however, as the concentration of AP21967 decreases, FRB-AsCas12a dissociates and, due to its Nuclear Localization Sequence (NLS), translocates to the nucleus. The details of RNP release from VLPs remain unknown.

#### 3.1.2 Preliminary considerations

1. First, it is important to choose an efficient crRNA for a target locus. Candidate crRNAs could be selected from the published literature or designed using online tools such as Benchling (https://www.benchling.com/), CHOPCHOP (https://chopchop.cbu.uib.no/), or others. We suggest to experimentally validate at least three crRNAs per target locus. For preliminary evaluation of crRNA activity, we recommend to use a combination of the pU6-crRNA plasmid together with the plasmid coding for non-modified AsCas12a without the FRB domain and deliver plasmids using the best method suitable for your target cells (lipofection, electroporation, *etc.*). In this protocol, we used crRNA targeting *CXCR4* as an example (Borovikova et al., 2024).
2. Clone the most efficient crRNA under the control of the CMV promoter. Generate plasmids for the expression of one crRNA or an array of three to six copies of crRNA under the control of the CMV promoter. One copy is cloned using annealed oligonucleotides, whereas three and six copies can be cloned using sequential cloning of annealed oligonucleotides, but the most convenient method is cloning of pre-synthesized DNA fragments (Borovikova et al., 2024). We showed that the highest editing levels were achieved with six crRNA copies in an array (Borovikova et al., 2024). Therefore, the plasmid with six copies of crRNA against *CXCR4* under the control of the CMV promotor is used in this protocol (Figure 3, lower construct).

The construct coding for crRNA under the control of the CMV promoter shows lower editing efficiency in transfection; therefore, it is not necessary to test it additionally once the efficiency of pU6-crRNA is confirmed.

3. Prepare plasmids with CMV-crRNA and FRB-AsCas12a. In our case, pCMV-crRNA additionally codes for a fluorescent protein mClover that can be used to monitor transfection and VLP transduction efficiency (Borovikova et al., 2024).
4. Obtain the packaging plasmid (which is based on HIV-1 in this protocol) and the VSVG plasmid.
5. To account for VLP toxicity and nonspecific effects, consider including an irrelevant non-targeting crRNA and prepare control VLPs with this crRNA.
6. Titrate VLPs to choose the highest editing dose that does not affect cell viability and growth.
7. Choose the method for the analysis of editing efficiency. The editing at a target locus is usually analyzed at the DNA level (using Sanger sequencing or NGS) or at the protein level (using flow cytometry or Western blotting). In this protocol, editing levels are analyzed using flow cytometry.
8. Consider time points for the analysis of editing efficiency. At the DNA level, it is usually performed after 2–4 days, whereas at the protein level, it should be determined experimentally in preliminary experiments with plasmid transfection. Depending on the rate of protein turnover, it may take 2–7 days or even longer.

### 3.2 VLP production in a 9-cm Petri dish with GenJect-39 transfection

This protocol provides instructions for producing NanoMEDIC VLPs with AsCas12a in the format of a 9-cm Petri dish. 293T producer cells are first transfected with a mixture of plasmids using a commercial lipid-based transfection reagent. VLPs are accumulated in the culture supernatant and then collected and concentrated by centrifugation in a microcentrifuge. In total, 10 mL of the supernatant yields 200 µL of the VLP preparation, which corresponds to 50-fold concentrated preparation and is enough for 1–2 transduction experiments in a 96-well plate format with VLP dilution and for WB analysis.

#### 3.2.1 Seeding producer cells (day 0)

Thaw 293T cells and passage them at least twice before seeding cells for transfection to ensure that the cells are healthy. Check 293T cells for *mycoplasma* contamination regularly. Do not allow the cells to overgrow.

1. Seed 3.2 × 10^6^ 293T cells in a 9-cm Petri dish in 10 mL full medium.
2. Grow cells for 16–24 h.

#### 3.2.2 Transfection (day 1)

3. Prepare plasmid DNA (plasmid constructs are shown in Figure 3) and calculate the required volumes of each plasmid according to Table 1 (9-cm dish, GenJect-39). The volume (V, µL) needs to be calculated based on plasmid concentrations. Calculate the sum of all plasmid volumes (X).

**TABLE 1:** Plasmid DNA amount used for transfection. The volume (V, µL) needs to be calculated for each transfection method based on the plasmid concentration.

#	Plasmid	Ratio	m, µg			V, µL
			9-cm dish		15-cm dish	
			GenJect-39	PEI/Ca-Ph	Ca-Ph	
1	pCMV-VSVG	1	0.19	0.76	3.306	​
2	pHLS-FKBP12-Gag	4.1	0.781	3.124	13.59	​
3	pCMV-FRB-AsCas12a	5.9	1.12	4.48	19.49	​
4	pCMV-mClover-6X4	13.2	2.506	10.024	43.6	​
​	Total, µg	​	4.6	18.4	80	X

The plasmid ratio is one of the most important parameters that affect the resulting editing activity of VLPs. In this protocol, the plasmid ratio was chosen based on the studies by Gee et al. (2020) and Hamilton et al. (2021) and is different from that described in our study by Borovikova et al. (2024). Hamilton et al. (2021) suggested to produce Cas-VLPs using 1 µg of the VSVG plasmid and 10 µg of the gRNA plasmid and varied the ratio of the Gag-Cas and Gag-Pol plasmids, the sum of which was 10 µg. Gee et al. (2020) produced NanoMEDIC VLPs using the following amounts of plasmids: 10 µg of FKBP12-Gag, 10 µg of FRB-Cas9, 10 µg of gRNA, 5 µg of VSVG, and 2 µg of Tat. We reasoned that the higher ratio of the gRNA plasmid used by Hamilton et al. would be beneficial for VLP activity since several studies have identified gRNA as a limiting factor for efficient editing (Gee et al., 2020; Hamilton et al., 2021; Hamilton et al., 2024; Banskota et al., 2022; An et al., 2024). Therefore, we used the ratio suggested by Hamilton et al. in this protocol with slight modifications. The ratio of FKBP12-Gag to FRB-AsCas12a was 4.1:5.9, corresponding to the 1:1 ratio for pmol amount, and was chosen as in the study by Gee et al. (2020). The amount of the crRNA plasmid was chosen for the pKS-U6-crRNA plasmid. When the plasmid was replaced with the pCMV-mClover-6X4 plasmid, the same ratio for pmol was preserved.

4. Prepare two microcentrifuge tubes (A and B) and add 250-X µL Opti-MEM to Tube A and 240 µL Opti-MEM to Tube B.
5. Add plasmid DNA to Tube A, vortex, and spin down.
6. Vortex GenJect-39, spin down, and transfer 9.2 µL to Tube B; pipette several times to wash the reagent from the tip into the solution, and then, mix by pipetting 3–4 times with a 1,000 µL pipette. Incubate for 5–10 min at room temperature (RT).

The ratio of DNA, µg to GenJect-39, µL at 1:2 was chosen according to the manufacturer’s instructions.

When a small number of samples are prepared, Step 5 can be followed during the incubation of GenJect-39 in Opti-MEM.

7. Transfer GenJect-39 from Tube B to Tube A dropwise and mix by pipetting 3–4 times with a 1,000 µL pipette. Incubate for 15–20 min at RT.
8. Add the DNA-GenJect-39 mixture to the dish with cells dropwise, spreading droplets around the dish. Swirl the dish gently and place into the CO_2_ incubator for 4–24 h.

We did not observe significant difference in the VLP yield when GenJect-39 transfection was left for 4 h or 16–24 h and therefore used either condition, whichever was most convenient.

9. Prepare 10 mL full medium in a sterile 50 mL tube and place it into the CO_2_ incubator to pre-warm it at 37 °C.
  Mark the tube “no AP21967” to avoid forgetting to add the dimerizer directly before medium change.

#### 3.2.3 Medium change [4 h later (day 1) or 16–24 h later (day 2)]

10. Add 10 µL AP21967 stock solution to the 9 mL medium prepared in Step 9 and mix. The final concentration of AP21967 will be 300 nM.

Since the AP21967 stock solution is in ethanol, keep it in a cooled rack to reduce its evaporation and add it to the pre-warmed medium directly before adding to cells. The final concentration of AP21967 is consistent with previously published data (Gee et al., 2020; Watanabe et al., 2023) and with our prior study, ensuring maximal RNP recruitment into VLPs (Borovikova et al., 2024).

11. Aspirate the medium from the dish and gently add the fresh medium with AP21967 to the side of the dish.
12. Place the dish into the CO_2_ incubator and incubate for 72 h if the medium is replaced on the same day or for 48 h if the medium is replaced after 16–24 h.

#### 3.2.4 VLP concentration (day 4)

For each VLP sample, prepare the following materials (in addition to the standard equipment in a flow hood):

- 15 mL tube *(one additional tube may be required—see explanation in Step 20)*
- 0.45 µm syringe filter
- 6-cm Petri dish
- 10 mL syringe
- 6× 1.5 mL microcentrifuge tubes

Set microcentrifuge at a “precool” program.

If VLPs and producer cells are required for WB analysis, prepare the following materials:

- 5 mL fresh full medium
- 1.5 mL tube

13. Using an epifluorescent microscope, check the signal of the fluorescent protein mClover encoded by the pCMV-mClover-6X4 plasmid. When illuminated using a blue laser, most cells should be green.
14. Transfer the cell culture supernatant from the Petri dish to the 15-mL sterile tube.

If producer cells are required for WB analysis, gently add 5 mL full medium to the dish and leave it in the incubator. They can be processed during VLP centrifugation (2.5 h) or later on the same day. If needed, producer cells can be covered with fresh full medium (minimum 5 mL), left overnight, and lysed on the next day.

15. Centrifuge the tube at 350 *g* for 5 min at RT to collect cellular debris.
16. Transfer the supernatant from the tube (∼9 mL) to the 6-cm Petri dish.
17. Take a syringe without the needle.
18. Collect the supernatant from the dish with the syringe.
19. Open the syringe filter and put it on the syringe.
20. Carefully filter the supernatant into six 1.5-mL tubes one by one.

Alternatively, the supernatant may be filtered into another 15 mL tube and aliquoted into 1.5 mL tubes with a pipette.

21. Place tubes into a precooled centrifuge and concentrate VLPs at 21,000 *g* for 2.5 h at 4 °C.

During this time, producer cells can be processed for WB analysis. See Section 3.7.

Before centrifugation is completed, place the DNAse I buffer from the refrigerator to the rack at RT.

Set the thermostat at 37 °C.

22. Take 1.5 mL tubes out of the centrifuge and place under the hood. A small whitish pellet should be visible at the bottom of each tube.

It is best observed at the white background and is convenient in a laminar flow hood with white interior. If a hood has gray interior, it may be difficult to observe the pellets.

23. Take a 1 mL pipette and aspirate supernatants from all six tubes, leaving ∼100 µL in each tube.
24. Take a 200 µL pipette and aspirate an additional volume of the supernatant from all six tubes, leaving ∼10–15 µL in each tube.
25. Add 50 µL Opti-MEM into one tube, pipette 5–8 times, transfer to the next tube, and pipette again. Repeat this procedure to resuspend all VLPs and combine them in one tube.
26. Measure the resultant volume with a 200-µL pipette.

A volume of 90–120 µL is usually obtained.

27. Adjust the volume with Opti-MEM to 180 µL. Measure with a pipette and mix by pipetting or gentle vortexing.
28. Optional (if VLPs and producer cells are required for WB analysis): transfer 21 µL of the VLP sample into a 0.5–1.5 mL tube and leave it at 4 °C for subsequent processing. Sample preparation for WB should be done on the same day—see Section 3.7; alternatively, freeze VLP aliquots and process them later.
29. Add the DNAse I buffer (1/9 of the VLP volume: 20 µL or 17.8 µL, if an aliquot was taken for WB).
30. Add 1 µL DNAse I and mix by pipetting 3–4 times. Spin down.
31. Incubate the tube at 37 °C for 10 min.

Omitting DNAse I treatment decreases editing efficiency.

32. Spin down and aliquot per 30–50 µL.
33. Store at −70 °C.

At least one freeze–thaw cycle does not affect editing efficiency of VLP preparation. Nevertheless, repeated freeze–thaw cycles should be avoided.

The resultant VLP sample can be used for transduction of target cells.

### 3.3 VLP production in a 9-cm Petri dish with polyethyleneimine (PEI) transfection

Seed 293T cell as described in Steps 1–2 of Section 3.2.1.

Before starting, take PEI from the refrigerator and allow it to thaw at RT.

3. Prepare plasmid DNA and calculate the required volumes of each plasmid according to Table 1 (9-cm dish, PEI/Ca-Ph) and the total volume of plasmids (X).

The amount of DNA needed for PEI transfection is ∼4-fold higher than that for transfection with GenJect-39, but the molar ratio between individual plasmids is preserved.

4. Prepare two microcentrifuge tubes (A and B), add 250-X µL Opti-MEM into Tube A and 176 µL Opti-MEM to Tube B.
5. Add plasmid DNA to Tube A, vortex, and spin down.
6. Vortex PEI, spin down, and transfer 73.6 µL to Tube B, pipette several times to wash the reagent from the tip into the solution, then mix by pipetting 3–4 times with a 1,000 µL pipette.

Several ratios of DNA, µg to PEI, µL should be evaluated, including 1:2, 1:3, 1:4, and 1:5. The ratio 1:4 produced the highest transfection level and was chosen for VLP production in our protocol.

7. Transfer PEI from Tube B to Tube A dropwise and mix by pipetting 3–4 times with a 1,000 µL pipette. Incubate for 30 min at RT.
8. Add the DNA–PEI mixture to the dish with cells dropwise, spreading droplets around the dish. Swirl the dish gently and place into the CO_2_ incubator for 6 h.

Perform Steps 9–33 described in Section 3.2.

### 3.4 VLP production in a 9-cm Petri dish with calcium-phosphate transfection

Seed 293T cells as described in Steps 1–2 of Section 3.2.1.

Before starting, take 2× HBS and 2M CaCl_2_ solutions from the refrigerator and allow them to thaw at RT.

3. Prepare plasmid DNA and calculate the required volumes of each plasmid according to Table 1 (9-cm dish, PEI/Ca-Ph) and the total volume of plasmids (X).

The amount of DNA needed for calcium-phosphate transfection is ∼4-fold higher than that for transfection with GenJect-39, but the molar ratio between individual plasmids is preserved.

4. Prepare two microcentrifuge tubes (A and B), add 438-X µL sterile deionized water, 62 µL 2M CaCl_2_, and plasmid DNA into Tube A, vortex, and spin down.
5. Add 500 µL of 2× HBS to Tube B.
6. Set the vortex to a moderate speed. Ensure that Tube B can be vortexed while open without spilling the liquid.
7. While vortexing, add the DNA solution from Tube A dropwise into Tube B. Incubate at RT for 30 min.
8. Add the DNA–calcium phosphate solution to the dish with cells dropwise, spreading droplets around the dish. Swirl the dish gently and place into the CO_2_ incubator for 6 h.

Perform Steps 9–33 described in Section 3.2.

### 3.5 VLP production in a 15-cm Petri dish with calcium-phosphate transfection

This protocol describes VLP production in a larger volume (×3) than that used in the protocol for a 9-cm Petri dish. Since commercial transfection reagents including GenJect-39 and its alternative lipid-based formulations are too expensive for transfection in large formats, this protocol uses calcium-phosphate transfection with larger amounts of plasmid DNA. To this end, we performed manual plasmid purification, which yielded more than 1 mg of plasmid DNA that could be resuspended at a high concentration. In total, 30 mL of the supernatant yields 600 µL of VLP preparation with 50-fold concentration, which is enough for several transduction experiments.

#### 3.5.1 Seeding producer cells (day 0)

1. Seed 9 × 10^6^ 293T cells in a 15-cm Petri dish in 30 mL full medium.
2. Grow cells for 16–24 h.

#### 3.5.2 Transfection (day 1)

Prior to transfection, take 2× HBS and 2M CaCl_2_ solutions from the refrigerator and allow them to thaw at RT.

3. Prepare plasmid DNA and calculate the required volumes of each plasmid according to Table 1 (15-cm dish, Ca-Ph) and the total volume of plasmids (X).
4. Prepare a microcentrifuge tube (Tube A), add 1,140-X µL sterile deionized water, 160 µL 2M CaCl_2_, and plasmid DNA, vortex, and spin down.
5. Prepare a 15-mL tube (Tube B) and add 1,300 µL 2× HBS.

Perform Steps 6–8 described in Section 3.4 and then Steps 9–12 described in Section 3.2 using 30 mL full medium and 30 µL AP21967.

#### 3.5.3 VLP concentration (day 4)

For each VLP sample, prepare the following materials:

- two ultracentrifuge tubes
- two 50 mL tubes
- 0.45 µm PVDF syringe filter
- 9-cm Petri dish
- 10 mL syringe

Set ultracentrifuge at a “precool” program (1,000 *g*, 2 °C, 1 h).
Spray ultracentrifuge tubes, adapters, and adapter caps with ethanol and dry inside the flow hood for sterilization.

13. Using a epifluorescent microscope, check the signal of the fluorescent protein mClover encoded using the pCMV-mClover-6X4 plasmid. When illuminated using a blue laser, most cells should be green.
14. Transfer the cell culture supernatant from the Petri dish to the 50 mL sterile tube.
15. Centrifuge the tube at 350 *g* for 5 min at RT to collect cellular debris.
16. Transfer the supernatant (∼28–29 mL) from the tube to the 9-cm Petri dish.
17. Take the syringe without the needle.
18. Collect the supernatant from the dish with the syringe.
19. Open the syringe filter and put it on the syringe.
20. Carefully filter the supernatant into the 50 mL tube.
21. Bring the total volume to 48 mL with PBS.
22. Transfer the supernatant into two ultracentrifuge tubes (24 mL per tube).
23. Sublayer 4 mL of 20% sucrose/PBS below the supernatant using a serological pipette with a controller.

When pipetting, leave a small volume of sucrose in the pipette (∼0.2–0.4 mL) not to disturb the layer above (serological pipettes usually take out the last drops of liquid with a splash).

24. Using flame-sterilized forceps, hold the tubes by the top edge, transfer them into the adapters, and close the adapters with caps.
25. Using a wide rack for 50 mL tubes that can accommodate the adapters for ultracentrifuge tubes, weigh the first adapter with the tube and tare the balance. Weigh the second adapter with the tube and record the resulting value. Return to the flow hood, open the caps, and add the corresponding volume of sterile PBS to the lightest tube. Place caps the caps and check the balance. Both adapters with tubes and caps should have equal mass.
26. Place the adapters with tubes into a precooled ultracentrifuge and concentrate VLPs at 112,400 *g* for 2 h at 1 °C.

Centrifugation parameters were as described for Cas-VLPs by Hamilton et al. (2021).

Ensure that the ultracentrifuge is balanced and starts to speed up.

Before centrifugation is completed, place the DNAse I buffer from the refrigerator to the rack at RT.

Set the thermostat at 37 °C.

27. Take the adapters with the tubes out of the ultracentrifuge and place under the hood.
28. Open the adapters and remove the tubes using sterilized forceps.
29. Since the walls of the tubes are slightly opaque, no apparent pellet can be observed at the bottom.
30. Take a 10 mL serological pipette and aspirate supernatants from both tubes, leaving ∼2–3 mL in each tube.
31. Take a 1,000 µL pipette and aspirate an additional volume of the supernatant from both tubes, leaving ∼20–50 µL in each tube.
32. Add 100 µL Opti-MEM to both tubes and pipette 5–8 times.
33. Cover both tubes with parafilm and incubate at 4 °C for 1 h.

This step should improve VLP resuspension.

34. Pipette the supernatant in both tubes and transfer into a single 1.5-mL tube.
35. Measure the resultant volume with a 1,000 µL pipette.
36. Adjust the volume with Opti-MEM to 540 µL. Measure with a pipette and mix by pipetting or gentle vortexing.
37. Add 60 µL 10× DNAse I buffer.
38. Add 3 µL DNAse I, mix by pipetting 3–4 times, and spin down.
39. Incubate at 37 °C for 10 min.
40. Spin down and aliquot per 30–50 µL.
41. Store at −70 °C.

The resultant VLP sample can be used for transduction.

### 3.6 Evaluation of VLP editing efficiency using flow cytometry

This protocol is performed to determine VLP editing efficiency. Target Jurkat T cells are transduced with serial dilution of VLPs, and *CXCR4* knockout levels are analyzed on day 6 after transduction using flow cytometry.

#### 3.6.1 Transduction of target cells (day 0)

An example for three VLP samples produced using three different transfection methods is provided. Transduction is performed in a flow hood.

1. Grow target cells (see the notes for 293T cells in Section 3.2.1).
2. Prepare a flat-bottom sterile 96-well plate and mark it according to Table 2. Do not use edge wells.
3. Prepare sterile microcentrifuge tubes for VLP dilution (five tubes per sample in this example) and mark them.
4. Transfer 75 µL full medium into each tube.
5. Thaw VLPs at RT, vortex gently, and spin down.
6. Prepare serial dilutions of each VLP sample: transfer 75 µL from the tube with the VLP stock preparation into the first tube with the medium, pipet 8–10 times, transfer to the next tube, repeat for all tubes according to Table 3.
7. Place the VLP dilutions at 4 °C, mark the VLP preparations as “used,” and place at −70 °C for later use (if needed).
8. Count Jurkat T cells. Calculate the required number of cells for transduction (0.45 × 10^5^ per well).

**TABLE 2:** VLP transduction scheme.

Sample VLP, µL	VLP#1 (GenJect-39)	VLP#2 (PEI)	VLP#3 (Calcium-phosphate)	Control samples
35	​	​	​	no VLP control
17.5	​	​	​	no VLP control
8.8	​	​	​	​
4.4	​	​	​	​
2.2	​	​	​	​

**TABLE 3:** VLP dilution scheme.

Sample VLP, µL	Tube №	VLP
35	1	75 µL full medium + 75 µL VLP preparation (stock)
17.5	2	75 µL full medium + 75 µL VLP from tube 1
8.8	3	75 µL full medium + 75 µL VLP from tube 2
4.4	4	75 µL full medium + 75 µL VLP from tube 3
2.2	5	75 µL full medium + 75 µL VLP from tube 4

The required number of cells, mln = 0.45 × 10^5^ × (5 dilutions × 3 VLP samples + 2 control samples) × 1.1.

9. Transfer the volume with the corresponding number of cells into a 1.5-mL tube.
10. Centrifuge cells at 350 *g* for 5 min.
11. Discard the supernatant and add the fresh full medium as follows:

The required volume of the medium, mL = 0.02 mL × (5 dilutions × 3 VLP samples + 2 control samples) × 1.1.

12. Resuspend cells and transfer 20 μL cell suspension into each well of a marked 96-well plate.
13. Transfer 70 µL VLP dilutions into corresponding wells. Pipette the suspension several times after VLP addition.

To reduce the usage of tips, we transfer each serial dilution (one VLP sample) with the same tip from the highest to the lowest dilution (from tube 5 to tube 1).

14. Add 70 µL fresh medium to control wells and pipette the cell suspension.
15. Place the plate in the CO_2_ incubator and leave it overnight.

#### 3.6.2 Medium change (day 1)

Incubation of cells with VLPs is performed in a small volume to increase transduction efficiency. However, this volume is not optimal for the cell growth. We suggest to add the fresh medium on the next day after transduction.

16. Add 110 µL fresh full medium to each well.
17. Resuspend the cells in each well.

This step is optional. However, cells grow faster, if they are resuspended more often, especially after transduction and when seeded in small quantity.

18. Place the plate in the CO_2_ incubator and leave it overnight.

#### 3.6.3 Medium change (day 2)

We observe that Jurkat T cells grow faster if the medium is changed more often. Therefore, we usually change the medium on day 2 after transduction. If inconvenient, this step can be omitted. In this case, add 200 µL medium in Step 16.

19. Remove 120 µL medium and resuspend the cells in each well.
20. Add 200 µL fresh full medium to each well.
21. Place the plate in the CO_2_ incubator and leave it overnight.

#### 3.6.4 Cell passage (day 4)

Cells should not overgrow. Therefore, we usually passage them on day 4. To obtain the number of cells enough for flow cytometry and genomic DNA extraction (optional), we transfer cells from a 96-well plate into a 48-well plate.

22. Mark wells in a 48-well plate and add 400 µL full media in each well.
23. Resuspend and transfer the entire volume of cells from a 96-well plate into the corresponding wells in a 48-well plate.
24. Place the plate in the CO_2_ incubator.

#### 3.6.5 Flow cytometry (day 6)

The day for knockout measurement should be determined empirically depending on the protein of interest. Day 6 was chosen in our study.

25. Prepare a 1.5-mL tube for the anti-CXCR4 antibody dilution (1:100) and cover it with the aluminum foil.
26. Calculate the volume of antibody needed as follows:
  (5 dilutions × 3 VLP samples + 2 controls) × 30 µL staining volume × 1.1 = X, µL PBS

X/100 = Y, µL antibody stock solution.

We use the antibody directly labeled with phycoerythrin. Alternatively, indirect labeling can be used.

27. Add X µL of PBS and Y µL of antibody stock solution into the tube, vortex, and spin down. Keep protected from light.
28. Resuspend and transfer 200 μL cells from each well into a round-bottom 96-well plate. Split each control sample into two wells (stained and unstained).
29. Prepare a counter balance and centrifuge the plate at 700 *g* for 3 min.
30. Aspirate the supernatant and resuspend the cells in 200 µL PBS with an 8-channel pipette.
31. Centrifuge the plate at 700 *g* for 3 min and aspirate the supernatant.
32. Resuspend the cells in two control wells in 30 µL PBS and the cells in all other wells in 30 µL antibody solution.
33. Incubate the cells at 4 °C for 30 min.
34. Wash the cells twice with PBS.
35. Resuspend the cells in 180 µL PBS and measure the level of CXCR4 using flow cytometry.

**FIGURE 4:**
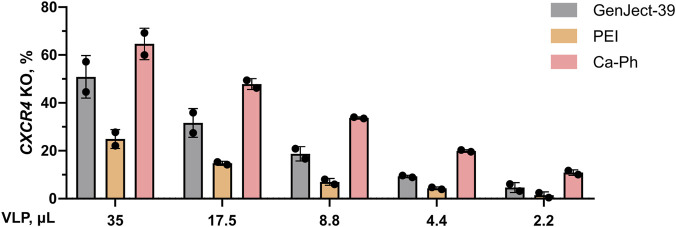
*CXCR4* editing efficiency of VLP preparations produced using three different transfection methods [GenJect-39, polyethyleneimine (PEI), and calcium-phosphate (Ca-Ph)]. Results of two independent transduction experiments of Jurkat T cells with the same VLP preparations are shown. Data are presented as mean ± standard deviation; individual values are plotted as dots.

Example results are shown in Figure 4.

### 3.7 VLP analysis using Western blotting

**FIGURE 5:**
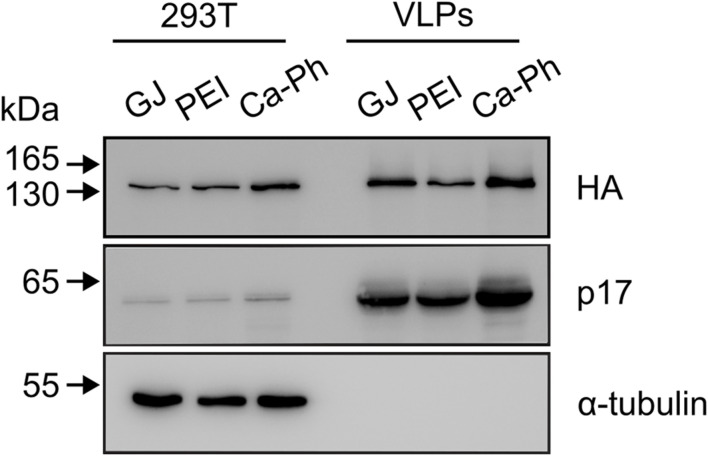
Western blot analysis of VLP preparations produced using three different transfection methods [GenJect-39 (GJ), polyethyleneimine (PEI), and calcium-phosphate (Ca-Ph)].

This protocol describes WB analysis of nuclease content in VLPs and producer cells. VLPs are lysed directly in the SDS-PAGE sample buffer. VLP producer cells are first lysed in the Triton X-100 buffer, and pellet-free lysates are heated in the SDS-PAGE sample buffer. Samples are subjected to SDS-PAGE, transferred onto a PVDF membrane, and probed with antibodies against HA tag (nuclease), p17 (Gag), and α-tubulin (a loading control for cell lysates, should be absent in VLP preparations). Example results are shown in Figure 5.

#### 3.7.1 VLP sample preparation for WB (day 0)

If preparation is done on the same day with VLP harvesting, take an aliquot of VLPs obtained earlier (see Section 3.2.4, Step 28). Alternatively, thaw an aliquot from −20 °C.

1. Thaw 4× SDS-PAGE sample buffer.
2. Add 7 µL of the 4× SDS-PAGE sample into each VLP sample (21 µL), mix, and incubate at 80 °C for 5 min.
3. Centrifuge samples and store at −20 °C.

#### 3.7.2 Preparation of VLP producer cells for WB (day 0)

4. Take VLP producer cells out of the CO_2_ incubator and put them under the flow hood.

Although samples for WB do not need to be sterile, all VLP experiments are performed under BSL2 conditions similar to the work with lentiviruses.

5. Wash cells with PBS and add 1.5 mL trypsin–EDTA solution to all dishes. Put into the CO_2_ incubator for 5–10 min.
6. Resuspend cells in one dish and deactivate trypsin by adding 5 mL full medium. Count cells using the cell counter or in a Goryaev chamber. Calculate the volume containing 1 × 10^6^ cells.
7. Resuspend cells and take an aliquot of 1 × 10^6^ cells into a 1.5-mL centrifuge tube.
8. Resuspend cells in the next dish, add 5 mL medium, and take the same volume of cells into a 1.5-mL centrifuge tube.
9. Repeat Step 8 with for all dishes.

The following suggestions are provided for an experiment using more than one 9-cm Petri dish:

- If VLP types and transfection methods are the same in all dishes, sample processing can be simplified. We assume that all dishes have an equal number of cells and count cells only in the first dish.
- If any of the parameters [VLP types (for example, NanoMEDIC and eVLP), transfection methods, DNA dosage, and/or culture conditions] varies between dishes, count cells in each dish because all mentioned conditions can affect cell growth.
- 293T cells are easily aggregated at a high density. Therefore, we do not recommend to deactivate trypsin in all dishes simultaneously. We usually leave dishes with cells in the trypsin–EDTA solution and process each dish one by one: resuspend in trypsin, add medium, count, and transfer an aliquot into a 1.5-mL tube.

10. Centrifuge tubes at 350 *g* for 5 min at RT.
11. Wash cells once with 200 µL PBS and place on ice.
12. Prepare a 1.5-mL tube with the cell lysis buffer (150 µL per sample × 1.1).
13. Add the PMSF stock solution into the cell lysis buffer (1.5 mL per sample × 1.1). Vortex, spin down, and keep on ice.

Take an aliquot of PMSF from the refrigerator just before use, incubate at RT for 5 min, and vortex. Ensure that all crystals are dissolved.

14. Resuspend each sample in 150 μL cell lysis buffer and place on ice.
15. Incubate for 15 min.

Set the microcentrifuge at a “precool” program.

16. Centrifuge samples at 12,000 *g* for 10 min at 4 °C.

During centrifugation, prepare 1.5 mL tubes by the number of samples and mark them; thaw 4× SDS-PAGE sample buffer and set the heating block at 80 °C.

17. Add 40 µL 4× SDS-PAGE sample buffer into each tube.
18. After centrifugation is complete, transfer 120 µL lysate supernatants into tubes with 4× SDS-PAGE sample buffer and vortex.
19. Incubate the samples at 80 °C for 5 min.
20. Centrifuge the samples and store at −20 °C.

VLP and producer cell samples are ready for SDS-PAGE. We usually pause at this step and perform electrophoresis later.

#### 3.7.3 SDS-PAGE (day 1)

Cast two resolving 7.5% gels with 5% stacking gels.

We prefer resolving and blotting samples for HA and p17 separately and in parallel since these signals overlay on a membrane, and stripping does not produce good quality images.

21. Thaw VLP and producer cell samples and dilute them 1:1 with 1× SDS-PAGE sample buffer: 15 µL sample +15 µL 1× SDS-PAGE sample buffer.
22. Set up a cassette in the Mini-RPOTEAN Tetra Cell, load 8 µL samples into each well of both gels according to Table 4 (the same scheme for both gels).

**TABLE 4:** VLP and producer cell sample loading for SDS-PAGE.

Markers	Producer cells			1× SDS-PAGE sample buffer	VLPs			1× SDS-PAGE sample buffer
​	GenJect	PEI	Ca-Ph	​	GenJect	PEI	Ca-Ph	​
5 µL	8 µL							

We suggest to leave an empty lane between the samples of producer cells and VLPs. This is because the signal in VLP preparations may be much higher than that in cell lysates and interfere with the signal in an adjacent lane with the cell lysate. Load 1× SDS-PAGE sample buffer in empty lanes to avoid edge effects and distortion of bands.

23. Run both gels at 75 V for 15 min and at 180 V for 40–60 min until bromphenol blue reaches the edge of the gel but does not leave it.

#### 3.7.4 Protein transfer (day 1)

24. Before electrophoresis ends, prepare 1× transfer buffer with ethanol, rinse the PVDF membrane with ethanol, and cut the blotting paper to the size of the membrane.

Adding 0.02% SDS to the transfer buffer can help blotting the FRB-AsCas12a fusion protein.

25. Open the gel cassette and rinse glass plates with distilled water.
26. Put the blotting membrane, the filter paper, and both gels into three plastic containers with 1× transfer buffer. Incubate them for 20 min on a shaker.
27. Set up transfer sandwiches in the Trans-Blot Turbo system (Bio-Rad) according to the manufacturer’s instructions and run the program at 25 V for 30 min (with constant voltage).
28. Incubate the membranes with blocking buffer at RT for at least 1 hour or leave it overnight at 4 °C.

#### 3.7.5 Membrane staining with antibodies (day 2)

29. Incubate the first membrane with primary antibodies against HA tag (1:3,000 dilution in 2.5 mL blocking buffer) at RT for 1 h in a roller shaker.
30. In parallel, incubate the second membrane with primary antibodies against p17 (1:3,000 dilution in 2.5 mL blocking buffer) at RT for 1 h in a roller shaker.
31. Wash three times with 0.1% Tween/PBS, with each wash lasting 10 min.
32. Incubate the first membrane with secondary antibodies against rabbit IgG (diluted 1:3,000 in 2.5 mL blocking buffer) at RT for 1 h in a roller shaker.
33. In parallel, incubate the second membrane with secondary antibodies against mouse IgG (diluted 1:3,000 in 2.5 mL blocking buffer) at RT for 1 h in a roller shaker.
34. Wash three times with 0.1% Tween/PBS, with each wash lasting 10 min.
35. Using a chemiluminescent reagent, develop the membrane and detect the signal using Imager.
36. Briefly wash the first membrane with 0.1% Tween/PBS and incubate with primary antibodies against α-tubulin (diluted 1:200 in 2.5 mL blocking buffer) at RT for 1 h in a roller shaker.
37. Wash three times with 0.1% Tween/PBS, with each wash lasting 10 min.
38. Incubate the first membrane with secondary antibodies against mouse IgG (diluted 1:3000 in 2.5 mL blocking buffer) at RT for 1 h in a roller shaker.
39. Wash three times with 0.1% Tween/PBS, with each wash lasting 10 min.
40. Using a chemiluminescent reagent, develop the membrane and detect the signal using Imager.

### 3.8 Data analysis and visualization

Plot data using GraphPad Prism software (GraphPad Prism 8.0.1 Software, USA), and prepare figures using Inkscape (https://inkscape.org/).

## 4 Results

### 4.1 The editing efficiency of VLPs produced using different transfection methods

The VLP production protocol described in this study was briefly outlined by our group earlier (Borovikova et al., 2024) and is based on GenJect-39-mediated lipofection in 9-cm Petri dishes. Although GenJect-39 is an efficient reagent that requires quite low amounts of plasmid DNA (∼4 µg per a 9-cm dish) and robustly produces high transfection levels, nevertheless, it is quite expensive for large-scale transfection. PEI and calcium-phosphate are cost-effective alternatives to GenJect-39 and are applicable for large-scale production of VLPs but require ∼4-fold higher amounts of plasmid DNA. Therefore, in this study, we extended the protocol to include three different transfection methods: GenJect-39, PEI, and calcium-phosphate. Figure 4 shows the representative results of *CXCR4* gene editing in the T cell line Jurkat with VLPs produced using these transfection methods. We found that calcium-phosphate transfection consistently produced VLPs with higher knockout levels across the whole range of VLP doses. The 35 µL VLP dose mediated ∼65% *CXCR4* knockout, while the same doses of VLPs produced with GenJect-39 and PEI demonstrated 50% and 25% editing levels, respectively. We cannot exclude that, after thorough optimization, PEI-mediated transfection could compete with GenJect-39 or calcium-phosphate transfection. At lower doses, calcium-phosphate transfection showed even higher editing efficiency than GenJect-39 and PEI. Therefore, we used calcium-phosphate transfection for VLP production at a large scale in 15-cm dishes.

### 4.2 WB analysis of VLPs

The efficiency of nuclease packaging upon VLP production can be analyzed using WB with samples of VLP producer cells and VLPs (Figure 5). In this study, we used FRB-AsCas12a with the 3xHA epitope tag that can be detected with α-HA antibodies. α-Tubulin was used as a loading control for cell lysates, whereas p17, a component of the HIV-1 polyprotein Gag, served as a loading control for VLP samples. p17 reflects the VLP yield, whereas HA shows nuclease content. For the same VLP type, the HA and p17 signals usually correlate. In addition, α-tubulin should be absent from the VLP preparation. Its presence would indicate contamination of the VLPs with the cytosol fraction.

As an example, the protocol was used to compare the AsCas12a content in producer cells and VLPs prepared using three different transfection methods. In VLPs, the highest levels of both nuclease and p17 were detected in VLPs produced using calcium-phosphate transfection, followed by GenJect-39 samples. This is consistent with the results of *CXCR4* editing in Jurkat cells (Figures 4, 5). In VLP producer cells, a slightly higher level of nuclease was observed with calcium-phosphate transfection, further supporting the results of editing experiments. The results of Western blotting analysis clearly show that editing efficiency of VLP preparations depend on higher expression of nuclease in the producer cells, efficient packaging of nuclease into VLPs, and higher VLP yield. In addition, the absence of α-tubulin in VLPs indicates no contamination with the cell debris.

### 4.3 The editing efficiency of VLPs produced in a 15-cm Petri dish

**FIGURE 6:**
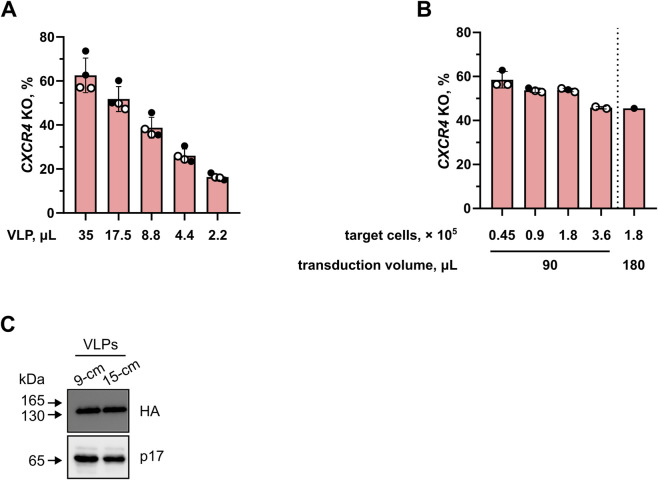
*CXCR4* editing efficiency of VLPs produced in a 15-cm Petri dish. Editing efficiency analyzed for VLP dilutions added to 0.45 × 10^5^ target cells **(A)** or 35 µL of VLPs added to the increasing number of cells **(B)**. Number of cells and transduction volumes are shown **(B)**. Two independent VLP preparations (black and white dots) were used in two independent transduction experiments (dots with the same color). Data are presented as mean ± standard deviation; individual values are plotted as dots. **(C)** Western blot analysis of VLP preparations produced in a 9-cm or 15-cm Petri dish using calcium-phosphate transfection.

To increase the VLP yield, we developed a protocol for a 15-cm Petri dish based on calcium-phosphate transfection. In contrast to the 9-cm dish protocol, the large-scale protocol includes ultracentrifugation of VLPs instead of centrifugation in a microcentrifuge because aliquoting large volumes of VLPs into multiple 1.5 mL tubes is impractical. A large-scale protocol produces 600 µL VLPs with ∼50-fold concentration. Comparable nuclease levels across both production scales were confirmed using WB analysis (Figure 6C).

The example result of transduction shows the *CXCR4* knockout level in Jurkat T cells for a series of VLP dilutions prepared with calcium-phosphate transfection in a 15-cm Petri dish (Figure 6). Editing results for two independent transduction experiments for each of the two independent VLP preparations are shown (Figure 6A). The observed editing efficiency was comparable (∼60%) to that obtained with the VLPs produced in a 9-cm Petri dish (∼65%); hence, the production of VLPs per mL of culture medium was not changed after scaling up. Figure 6B shows the editing efficiency of the same 35 µL VLP dose added to different numbers of target cells. This result indicates that cell numbers used for transduction can be increased at least 4-fold without losing VLP editing efficiency. This is important for subsequent scaling up of the protocol. Increasing the transduction volume from 90 to 180 µL slightly decreased editing efficiency (the last bar in Figure 6B).

## 5 Discussion

Here, we provided a detailed protocol for AsCas12a-VLP production originally described in our study by Borovikova et al. (2024). The protocol has several **advantages:**

- A detailed step-by-step protocol with commentaries on critical steps that could be helpful for researchers without prior experience in producing VLPs.
- Provides three alternative transfection methods, including those that are based on the use of cost-effective reagents and plasmids purified using home-made buffers.
- Describes two scales of VLP production, allowing adjusting the VLP yield depending on experimental needs.
- A typical VLP preparation at a laboratory scale is sufficient for several cell culture experiments with VLP dilutions.
- Applicable for comparison of different VLP types and VLP analysis using WB.

We believe that this protocol article depicts a validated and reproducible protocol successfully used for editing of several loci, including an endogenous locus in the T cell line Jurkat (Borovikova et al., 2024).

The **limitation** of the protocol is that it needs further large-scale optimization for preclinical settings. This would include stable cell line production and protocol optimization for the serum-free medium. Additionally, preclinical translation requires VLP characterization beyond standard WB and ELISA techniques. These include physical characterization by nanoparticle tracking analysis and transmission electron microscopy, stoichiometric quantification of nuclease loading by ELISA assays for Gag and nuclease, and high-throughput analysis of total protein and RNA content in VLPs by mass spectrometry and RNA-seq, respectively.

The described protocol utilizes the AsCas12a nuclease, which offers distinct advantages compared to the most widely used SpCas9. Although SpCas9 requires 5′-NGG-3′ PAM, AsCas12a recognizes an AT-rich 5′-TTTV-3′ PAM, thus enabling targeting of loci inaccessible to SpCas9. An additional advantage of AsCas12a is its inherent crRNA processing activity, which facilitates straightforward adaptation for multiplex genome editing. Furthermore, Cas12a orthologues are regarded as more specific than SpCas9 (Kim et al., 2016; Strohkendl et al., 2018; Baranova et al., 2025). It has been hypothesized that Cas12a is particularly suited for knock-in applications due to its distal cleavage pattern and the generation of staggered ends (Safari et al., 2019). Despite these strengths, the lack of Cas12a-based advanced genome editing tools, such as base and prime editors, currently limits further development and clinical translation of AsCas12a-based VLPs. However, the development of these Cas12a-based genome editing tools is ongoing.

Several **critical parameters** should be considered for the successful application of the protocol.

1. Prior to production of VLPs, it is necessary to select the most efficient crRNA. We recommend to test several candidate crRNAs in transient transfection of targets cells using plasmids for expression of crRNA under the control of the U6 promotor. Only when the efficient editing is proved with the U6-plasmid, crRNA should be used for VLP production.
2. VLP producer cells should be in the logarithmic growth phase and mycoplasma-free.
3. Transfection efficiency and the quality of plasmids and cells should be evaluated.
4. Ensure not to aspirate all supernatants after VLP centrifugation. We observed that discarding all supernatants after centrifugation (either in a microcentrifuge or in an ultracentrifuge) drastically decreases the VLP yield; therefore, we always leave a small volume of the medium above the pellet.
5. The treatment with DNAse I is important for VLP activity.
6. Perform transduction in a small volume (90 µL in this protocol) overnight. Add the fresh medium on the next day.
7. The number of target cells affects editing efficiency. We showed that VLP editing efficiency is equally high for cell numbers between 0.45 × 10^5^ and 1.8 × 10^5^. However, it should be tested experimentally for other cell types. Start with the smallest number of cells that can reliably proliferate in a 96-well plate.
8. Cell growth in a 96-well plate can be increased by a more frequent medium change and cell resuspension.
9. The time point for the analysis of knockout by flow cytometry should be tested for every target protein. Harvesting cells too early may lead to incomplete knockout presentation. Protein turnover should be considered, and the optimal time point should be determined experimentally. It may take 2–7 days or even longer.
10. Before proceeding with a genome editing experiment in a cell line of interest, we suggest to perform test experiments on VLP editing efficiency in 293T cells since these cells are efficiently transduced with VSVG-pseudotyped VLPs. If the protein of interest is not present in 293T cells, the editing activity could be analyzed at the level of genomic DNA using NGS or Sanger sequencing. For test experiments, we recommend Sanger sequencing, followed by the analysis of the indel level using the online tools, such as ICE (https://ice.editco.bio/) or TIDE (https://apps.datacurators.nl/tide/).

If no or a very low editing level is observed after all steps above are completed, VLPs should be analyzed using WB to confirm their presence in the preparation. If they are not detected, some steps in the protocol must be reconsidered.

11) When efficient editing with VLPs is demonstrated in 293T cells, we recommend to evaluate the transduction efficiency of a cell line of interest with VSVG-pseudotyped lentivirus expressing a fluorescent protein reporter. If the cell line is not transduced effectively with such lentivirus, then it is not likely to detect VLP-mediated genome editing in this cell line. For example, we observed this effect in the CEM cell line that had a lower transduction level than Jurkat T cells and was not edited by VLPs in our experiments. To address this issue, another envelop for efficient transduction of target cells should be considered. It should be investigated whether there are some specific envelopes for your cells of interest. For example, the BaEVRLess/VSVG combination was successfully used for lymphocytes (Gutierrez-Guerrero et al., 2021), and other envelopes are being explored (Banskota et al., 2022; Hamilton et al., 2024; Nielsen et al., 2024).

### 5.1 Additional considerations

**Applicability to other VLP types and other nucleases:** The described protocol is applicable not only to AsCas12a-VLPs based on the NanoMEDIC packaging mechanism but also to VLPs with SpCas9 and other VLP types since most procedures are not specific to AsCas12a or NanoMEDIC VLPs. The only feature of this protocol that is based on the characteristic of AsCas12a and is not directly transferable to SpCas9 is the usage of CMV-driven crRNA. The similar strategy to improve the packaging of SpCas9 gRNA into VLPs by its Pol II-driven expression and cytoplasmic localization is impossible because SpCas9 does not have pre-crRNA processing activity. One alternative approach was suggested by Gee et al. (2020) for NanoMEDIC VLPs, where gRNA flanked by ribozymes was encoded in an LTR-driven transcript with the ψ packaging signal of HIV-1. Although the ψ packaging signal was not present in our plasmid, the transcript with gRNA and ribozymes was inefficient in our hands (Borovikova et al., 2024). Recently, Pol II-dependent expression of pegRNA for the prime editor was applied in Nanoscribes VLPs by flanking it with Csy4 cleavage sites and adding a plasmid for this endonuclease into the VLP production mixture (Halegua et al., 2025). This approach may be tested with our protocol in subsequent experiments.

**Analysis of editing at the protein or DNA level:** In this protocol, VLP editing efficiency (the level of *CXCR4* knockout) is analyzed using flow cytometry. In cases, where it is inconvenient due to the lack of antibodies, inefficient intracellular staining, or other reasons, genomic DNA can be extracted from target cells (day 2–4), and indel frequency can be determined using the analysis of indel levels (for instance, ICE analysis). However, analysis of knockout at the protein level is most accurate if functional studies with the cells of interest are planned since not all the indels lead to protein loss. Therefore, we suggest to measure *CXCR4* knockout at the protein level using flow cytometry, which is a high-throughput method and provides single-cell resolution in contrast to WB.

**Number of replicates:** Normally, for optimization steps, we recommend producing two independent VLP preparations and testing each of them in transduction. When a large number of VLP variants is produced and compared, at least two replicates are needed (consider your resources and time for each locus/cell type/VLP type). We recommend always sticking to a minimum of three replicates.

**VLP titration:** We recommend always titrating VLP samples and comparing VLPs at several dilutions (at a 1:2–1:4 dilution ratio) since the difference between VLP types may be better observed at higher dilutions. Moreover, it is important to determine the lowest dose that still produces editing levels high enough for downstream applications. This will help reduce VLP use and increase target cell numbers with the same amount of VLP preparation.

**Application of WB for VLP analysis:** WB analysis of the nuclease content in the VLP samples shows the presence of the nuclease and its integrity (or degradation). More information can be obtained by comparing the nuclease content between VLPs and VLP producer cells. If only one VLP sample is analyzed, this comparison helps confirm the expected outcome: the molecular weight of nuclease is the same in producer cells and in VLPs (for NanoMEDIC particles) or it is reduced as the Gag–nuclease fusion undergoes protease cleavage [for eVLP and Cas-VLPs (Hamilton et al., 2021; Banskota et al., 2022)]. When several VLP types are used, this comparison allows to analyze different nuclease packaging mechanisms and choose the most efficient. The signals of the nuclease from VLP producer cells can reveal the nuclease fusions that are poorly expressed or degraded in the cell, while a side-by-side comparison of producer cells and VLPs can indicate what packaging mechanism provides the best nuclease enrichment in VLPs. Therefore, we suggest to always include samples of VLP producer cells into WB analysis of VLPs.

**FIGURE 7:**
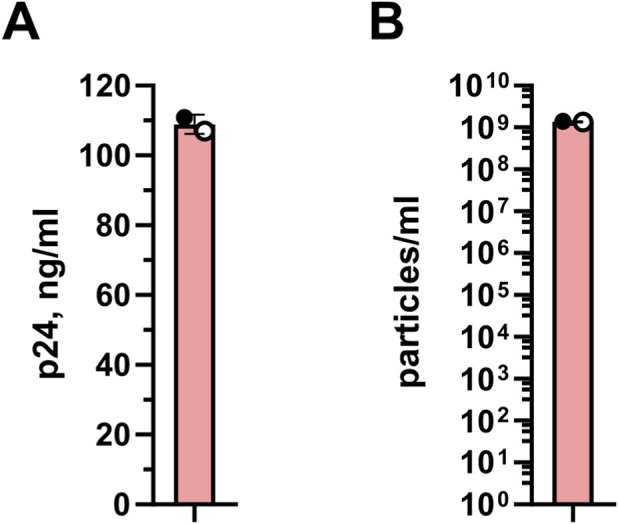
The p24 content **(A)** and the particle number **(B)** in VLPs prepared in a 15-cm dish. Data are presented as mean ± standard deviation; individual values are plotted as dots. Two independent VLP preparations are shown as black and white dots. The number of VLP particles/mL is calculated based on two figures: there are 2,000 p24 molecules per particle, and the mass of p24 is 24 kDa.

**Evaluation of the VLP count:** The amount of VLP particles in a preparation can be roughly estimated using ELISA to detect p24 (the HIV-1 capsid protein present in VLPs). We measured the p24 content and the corresponding number of particles in VLPs prepared in the 15-cm dish using a commercial kit (#0134, Vector-Best, Russia) (Figures 7A,B). The calculation of the VLP particle number by p24 is based on the assumption that VLPs contain the same number of p24 molecules as HIV-1 viral particles, which is estimated and usually assumed to be approximately 2,000 molecules, although various numbers have been determined (Briggs et al., 2004; Lavado-García et al., 2021; Kuzmichev et al., 2023). However, this is probably not exactly the case, and different VLP types may have slightly different p24 contents. Additionally, some ELISA kits differentiate between the VLP-bound and free protein, and some do not (Bystryak and Santockyte, 2015). Moreover, the p24 content is impossible to correlate with editing efficiency for different crRNAs and cell types. Therefore, the most important parameter that should be analyzed first is the editing efficiency for the given volume of VLPs. Together with the information on the degree of VLP concentration, this will allow easier comparison between studies. In our opinion, p24 ELISA has practical importance only for large-scale VLP preparations after all preliminary experiments and optimizations have been performed and studies with primary cells are required. For primary cells that have strict culturing conditions and where VLP titration is impossible to perform in advance, p24 measurement in a new preparation can help determine the required VLP dose.

**The degree of the VLP concentration:** VLP concentration by ∼50-fold used in this protocol was empirically chosen. For different primary cell types and animal models, a higher degree of concentration will be probably required. In some studies, VLPs were concentrated by 100-fold for editing of primary human cells (Gee et al., 2020; Banskota et al., 2022). For studies in animal models, NanoMEDIC VLPs were concentrated by 480-fold (Gee et al., 2020), and eVLPs with ABE were concentrated by 1,000–3,000-fold (Banskota et al., 2022).

**FIGURE 8:**
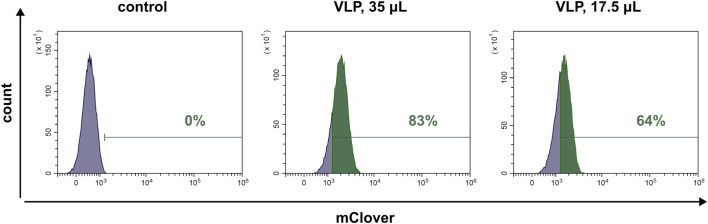
mClover fluorescence in target cells measured 2 days after VLP transduction.

**Evaluation of VLP transduction efficiency:** Some VLP types, such as eVLPs and Cas-VLPs, contain viral enzymes and thus can package, reverse-transcribe, and integrate a reporter, which was shown for Cas-VLPs (Hamilton et al., 2021). The reporter can be used to monitor transduction efficiency. In the case of NanoMEDIC VLPs that do not contain viral enzymes, the addition of a reporter is not applicable. However, our VLPs contain mClover that is expressed from the crRNA plasmid and is packaged passively. The fluorescent signal can be assessed using flow cytometry on day 2–3 after transduction and reflect the efficiency of VLP transduction of target cells (Figure 8). This can be used for troubleshooting, in addition to the steps mentioned above as the critical parameters.

**Pseudotyping:** In this study, we utilized the VSVG envelope due to its well-characterized, pantropic nature and its widespread use for both lentiviral and VLP-mediated delivery. VSVG pseudotyping serves as a critical benchmark for characterizing VLP editing efficiency before exploring targeted delivery approaches. These alternatives include envelopes from other viruses or hybrid systems incorporating receptor-blind VSVG mutants and a membrane-anchored antibody (Hamilton et al., 2024; Nielsen et al., 2024). The choice of envelope determines not only the specificity of delivery and functional VLP titers but also the location and biochemical conditions for the fusion between cellular and VLP membranes. For instance, VSVG mediates fusion inside endocytic vesicles upon their acidification (Kim et al., 2017), whereas alternative pseudotyping strategies may rely on other mechanisms and entry kinetics (Nikolic et al., 2018; Sharma et al., 2023). Such variations likely influence RNP release, stability, nuclear transport, and overall editing efficiency, although data on this area are currently limited. Overall, transitioning to alternative envelopes necessitates a re-evaluation of VLP production yields and requires further optimization of the preparation protocol, including plasmid ratios and the required level of the VLP concentration.

**Time considerations:** Starting from a 6-cm Petri dish of 293T cells recovered from thawing, the protocol takes ∼12–19 days, which includes 1) cell growth—0–7 days depending on the number of culture dishes, 2) VLP production—5 days, 3) WB—2 days (can be performed during cell cultivation after transduction), and 4) transduction and flow cytometry—6 days (for CXCR4).

## Data Availability

The original contributions presented in the study are included in the article/supplementary material; further inquiries can be directed to the corresponding author.
